# Leave no one behind: why WHO's regional office for Europe should prioritise children and adolescents in their program of work. A position statement from the European academy of paediatrics

**DOI:** 10.3389/fped.2023.1184870

**Published:** 2023-06-14

**Authors:** Danielle Jansen, Maria Brenner, Károly Illy, Łukasz Dembiński, Stefano del Torso, Zachi Grossman, Arunas Valiulis, Ann De Guchtenaere, Artur Mazur, Liviana Da Dalt, Ketil Størdal, Berthold Koletzko, Adamos Hadjipanayis

**Affiliations:** ^1^ Department of Primary and Long-term Care, University of Groningen, University Medical Center Groningen, Groningen, Netherlands; ^2^ Department of Sociology, University of Groningen, Groningen, Netherlands; ^3^ Accare, University Centre for Child and Adolescent Psychiatry, Groningen, Netherlands; ^4^ School of Nursing, Midwifery & Health Systems, University College Dublin, Dublin, Ireland; ^5^ Dutch Paediatric Society, Utrecht, Netherlands; ^6^ Department of Pediatrics, Hospital Rivierenland, Tiel, Netherlands; ^7^ European Academy of Paediatrics (EAP), Brussels, Belgium; ^8^ Department of Pediatric Gastroenterology and Nutrition, Medical University of Warsaw, Warsaw, Poland; ^9^ ChidCare WorldWide CCWWItalia OdV, Padova, Italy; ^10^ Department of Pediatrics, Maccabi Health Care Services Pediatrics, Tel Aviv, Israel; ^11^ Adelson School of Medicine, Ariel University, Ariel, Israel; ^12^ Clinic of Children’s Diseases, Institute of Clinical Medicine, Vilnius University Medical Faculty, Vilnius, Lithuania; ^13^ Department of Paediatrics, Ghent University, Ghent, Belgium; ^14^ Department Pediatrics, Pediatric Endocrinology and Diabetes, Medical College, University of Rzeszow, Rzeszow, Poland; ^15^ Department of Women’s and Children’s Health, University Hospital of Padova, Padova, Italy; ^16^ Department of Pediatric Research, Faculty of Medicine, University of Oslo, Oslo, Norway; ^17^ Division of Pediatric and Adolescent Medicine, Oslo University Hospital, Oslo, Norway; ^18^ Department of Paediatrics, LMU—Ludwig-Maximilians-Universität Munich, Division of Metabolic and Nutritional Medicine, Dr. von Hauner Children’s Hospital, University Hospital, LMU Munich, Munich, Germany; ^19^ Medical School, European University Cyprus, Nicosia, Cyprus

**Keywords:** children, adolescents, mortality, mental health, migrants, equity, world health organization, Europe

## Abstract

Children and adolescents are no longer a priority in the most recent European Programme of Work (EPW) 2020–2025 of the World Health Organization (WHO) Regional Office for Europe. In this position statement we provide arguments for why we think this population should be explicitly addressed in this important and influential document. We firstly emphasize the persistent health problems and inequalities in access to care for children and adolescents that are challenging to solve, and thus require a continuous focus. Secondly, we urge the WHO to prioritize children and adolescents in their EPW due to the new and emerging health problems related to global issues. Finally, we explain why permanent prioritization of children and adolescents is essential for the future of children and of society.

## Introduction

Children and adolescents are no longer a priority in the new European Programme of Work (EPW) 2020–2025 of the World Health Organization (WHO) Regional Office for Europe. The aim of our position statement is to provide arguments for why the WHO should continue to devote specific effort and resources towards improving the health of children and adolescents while being responsive to emerging child health priorities.

The EPW 2020–2025 of the WHO Regional Office for Europe shapes the European Region's contribution to the WHO's 13th General Programme of Work (1). In this EPW—“United Action for Better Health”, three core priorities are presented, accompanied by a vision of how the WHO Regional Office for Europe can support health authorities in Member States to meet these priorities. These priorities are: moving towards universal health coverage (UHC), protecting against health emergencies and promoting health and well-being. The importance of these priorities is, among other things, emphasized by their anchoring in the 2030 Sustainable Development Goals (SDGs) agenda.

The previous Programme, *Health2020: a European policy framework supporting action across government and society for health and well-being,* was agreed upon by all 53 Member States of the WHO European Region in 2012 (2). Similar to the current work programme, Health2020 presented priorities and provided politicians and policy practitioners with the main values and principles needed to put Health2020 into practice, and described leadership priorities of particular relevance for child and adolescent health.

Although aimed at the entire population in the European Region, Health2020 was full of texts and intentions to improve the health of children and adolescents specifically. The text is visionary and inspiring for every politician, policy maker, health care professional and community who wishes to grasp new opportunities to take the health and well-being of children and adolescents across Europe to a higher level. In the context of Health2020, specific plans were adopted by the WHO Regional Committee for Europe for child and adolescent health. A key strategy, *Investing in children: the child and adolescent health strategy for Europe 2015–2020* (3), was unanimously endorsed by the 53 Member States of the European region and aimed to enable children and adolescents in the WHO European Region to realize their full potential for health, development, and well-being, and to reduce their burden of avoidable disease and mortality.

Considering the significant and important emphasis placed on the needs of children and adolescents in Health2020, it is remarkable, and an immense opportunity missed, that this population is not explicitly addressed in the most recent EPW. In the 56-page document, the word child appears only four times and the word adolescent only twice. And although it says that “*the programmes on child and adolescent health and development should be reviewed*”, practice shows that since the release of the child and adolescent health strategy for Europe 2015–2020, there has been so far no visible intention to a follow-up strategy on child and adolescent health in the European region, to guide Member States further on towards the SDG goals 2030.

## Persistent health problems and inequalities in access to health care and health

Our first argument for prioritizing children and adolescents in the new EPW, concerns the fact that many health problems and inequalities in access to care remain for children and adolescents. Persistent health problems, spread over several SDG target areas, are challenging to solve and require a continuous focus from the WHO. The health of children and adolescents should remain a constant priority to encourage continual attention to the health and well-being of every child and adolescent in every country of the European region in the coming years and decades.

In 2019, when children and adolescents were deemed a high priority within the WHO, the WHO recognized the following challenges in the WHO European region: (1) large disparities in mortality rates of children under five years, between and within countries, with more than half of the deaths in children under five in the European region attributed to preventable and treatable causes; (2) considerable disparities in vaccine uptake; and (3) a high prevalence of overweight and obese children (4). For adolescents in the European Region, WHO specifically highlighted (1) the high suicide rates; (2) disparities in alcohol use; and (3) high prevalence of unsafe sex in some countries. These challenges all remain and, in many cases, have become even more severe since 2019. Here we discuss some specific examples.

### Under 5 mortality rates: many deaths due to preventable and treatable causes and large disparities between and within countries

When the WHO identified the mortality rate in those under 5 years as a focus area in 2019, the average mortality rate for this age group in the WHO European Region was 8 deaths per 1,000 live births and 4 neonatal deaths per 1,000 live births (5). Although this average rate was and still is the lowest rate globally (5), it still means that more than 160,000 children in the Region die before their fifth birthday every year. Mortality rate among young children is a closely monitored indicator because it reflects the access of children and populations to basic health services such as vaccinations and adequate nutrition and the quality of care they receive (6). As an example, recent reviews on nutrition, and nutrition related health, among children and adolescents in WHO's European Region (7, 8) showed that several nutrition issues, such as micronutrient deficiencies or underweight, are still significant and universal problems. In the Eastern European and Central Asian areas of WHO's European Region, anaemia is a particular nutrition challenge, with more than a fifth of adolescents affected (8). In addition, the prevalence of being underweight was, and is still, high (overall, around 8%–9%), especially in some Eastern European countries (8). Summarized, these results show that nowadays there remain significant nutritional issues that, despite being preventable, continue to be prevalent in the European region and contribute to the mortality of children under 5 years, particularly within certain countries and communities.

It is possible that this issue is not highlighted in the current EPW as there is considerable heterogeneity across the WHO European Region as the WHO highlighted: “*Although the European Region includes countries with the lowest number of infant and child deaths in the world, it also includes countries where children are 25 times more likely to die before the age of 5*” (4). It would raise grave concerns around equitable access to healthcare if heterogeneity as a utilitarian argument was acceptable for not continuing to highlight this issue given the ongoing preventable and treatable causes which underpin the mortality rate of children under 5 years. The virtually unchanged data indicates that efforts to reduce this mortality rate, as well as to reduce inequality between countries, remain essential.

### High prevalence of overweight, obesity, unsafe sex, and suicide

The second challenge for children and adolescents in the European region, as identified by WHO, is the high prevalence of overweight, obesity, unsafe sex, and suicide. In their 2020 review of school-aged child and adolescent nutrition in Europe and Central Asia region (7), UNICEF shows that, for boys and girls, respectively fourteen and ten countries had an overweight and obesity prevalence of ≥20%, while, in the case of boys, in three countries reached over 30%. More recently, the WHO European Regional Obesity Report 2022 (9) concluded that overweight and obesity affect nearly one in three children (29% of boys and 27% of girls) in the WHO European Region and that the prevalence increased during the COVID-19 pandemic. The assumption that overweight and obesity prevalence is increasing rather than decreasing, now and probably also in the future, is plausible given the increasing obesogenic environments and problematic digital marketing to children (9). A high prevalence of childhood overweight and obesity will lead to multimorbidity and complex multimorbidity in adult life (e.g., cardiovascular disease and cancer) and subsequently to high health care utilization and productivity loss, leading to a high societal burden in terms of costs (10). Regarding risky sexual behaviour (unsafe sex) the figures also remain worrisome. The most recent Health Behaviour in School-aged Children (HBSC) study reports that a quarter of sexually active 15-year-olds (one in four boys and one in seven girls) used neither condoms nor pills at their last sexual intercourse (11). Finally, the high prevalence of suicide in young people across the European region is still a huge public health problem and challenge to this day, especially since the years of the COVID-19 pandemic (12). In Europe, suicide is the second cause of death among adolescents aged 15–19: every year, nearly 1,200 European children and adolescents end their lives because of mental health difficulties (13). These facts are supported by concerns expressed by ministries of 48 countries in the WHO European region. Over 80% considered adolescent mental health, overweight and obesity as problem areas (14).

### Considerable disparities across countries

A further challenge that the WHO highlighted in 2019, and that is even more relevant today, is the striking differences across countries in the European region in vaccine uptake and health behaviour. Vaccines are among the most cost-effective interventions to reduce the burden of avoidable disease and mortality in young people (15). Yet, each year approximately 1 million children in the European Region do not receive all their scheduled vaccinations (4). Disparities in vaccine uptake were rightly a priority within the WHO: full childhood immunization coverage was and still is lower among certain populations compared to the general population or national average (16). The prevalence of alcohol use remains high and also varies greatly across countries and regions and across income groups (11). The results of the most recent HBSC study showed that the proportion of adolescents who had ever consumed alcohol varied from 2% of 11-year-olds adolescents (Kazakhstan) to 85% of 15-year-olds (Greece). Current alcohol use varied from 0.5% of 11-year-olds (Greenland and Ireland) to 67% of 15-year-olds (Denmark). A higher prevalence of alcohol use was reported in more affluent adolescents, probably because of greater accessibility and affordability (11).

## New and emerging health problems

Our second argument for prioritizing children and adolescents in the new EPW, concerns the new and emerging health problems. There are many issues that affect children and adolescents in the European Region and in the spring of 2022, UNICEF and WHO warned of the consequences of “*a toxic combination of growing poverty, inequality, conflict, climate change and COVID-19*” for the health of children and adolescents living in the European Region (17). While this is not only related to migration and war-related displacement, we currently face unusually high numbers of refugee children and their families applying for permanent or temporary protection in Europe, in addition to the large numbers of children and young people escaping Ukraine. Many refugee children may be unvaccinated and have physical health problems that might be common in the country of origin (for example, anaemia, chronic hepatitis B, latent tuberculosis), but rare in the host country. In addition, a significant proportion of refugee children also experience post-traumatic stress disorder, depression or anxiety disorder due to factors related to forced migration (18).

## What would be the benefits of keeping children and adolescents as a priority?

Our third argument for the need for permanent prioritization of the health of children and adolescents is its societal impact. In December 2021, the population of children 0–18 years old was 2.6 billion, accounting of one third of the global population (19). Childhood is a critical time in the development of human beings, and it is period when biological and behavioural determinants of life-long health are set, i.e., a golden opportunity for investing in health promotion and disease prevention of populations (20). The wellbeing of this large age group is thus vital for the development of societies, the world's future and for achieving the SDGs. Ensuring the best conditions in early life is, in the long term, the basis for a more productive and socially integrated adult individual. Investing in children's health is justified not only because it meets a basic human right, but also because it is an investment with high social and economic returns.

Vaccines are an excellent example to show how important it is that the WHO keeps investing in and prioritizing children and adolescents. As we all know, vaccines save lives. At the same time, vaccines are a wise economic investment. By investing very little, many diseases and disabilities that last a lifetime are prevented. Thus, millions of euros in potential healthcare spending by individuals and the health care systems are saved. Vaccines can also prevent the loss of salaries and reduce productivity loss due to illness and death, fostering economic growth. A study by Ozawa et al. (21) showed that projected immunizations will yield a net return about 16 times greater than costs over the decade. A recent study by Sim et al. (22) showed a very high return of investment from immunization against 10 pathogens. The study revealed that the return on investment was 51.0 from 2011 to 2020 and 52.2 from 2021 to 2030. The Decade of Vaccine Economics study (23) showed that from 2021 to 2030, every US $1 invested in vaccine programmes will avert around $20 in healthcare costs, lost caregiver wages and missed work, and lost productivity.

Adolescence is a crucial phase of a child's development to adulthood and, as shown before, a phase with many potential risks to health with fatal consequences in adulthood (24), including: tobacco and alcohol use, physical inactivity or intense physical activity, poor diet, overweight and obesity, mental disorders, and increase likelihood of contracting a sexually transmitted disease. Zhang et al. (25) estimated that in China the health expenditure per adolescent is only 85 dollars compared with health spending that is $382 per person. Mental disorders are a major cause of morbidity and mortality in adolescence. It has been estimated that 50% of lifetime mental disorders appear before the age of 14% and 75% by the age of 24 (26). An economic modelling study to quantify the potential costs and benefits of mental health interventions to prevent or treat mental disorders among adolescents showed a return on investment of 23,6 (27).

## Discussion

The examples referred to above provide clear evidence of the need for a specific focus on the health and health care of children and adolescents in Europe. In 2019 the viewpoint from Europe was that the quality of healthcare delivered to this cohort was less than optimal (28–30), and the current European programme of work of the WHO highlights a lack of impetus to address this (1). It is a lost opportunity to give a voice to the needs of the next generation.

Within this group are an increasing number of the *first-generation* of young people with integrated and complex care needs (31), many of whom are living longer than previous generations due to cutting-edge care delivery and advances in medical care. However, at a time when these young people are living longer, the current WHO EPW is choosing to ignore their emerging needs, thereby denying a focus on the structures and supports they need to become independent and autonomous citizens. This is critical for this group of young people given the specific need for their development of self-determination to cope with the many decisions and challenges they face. This is not confined to their health and social care needs, which can include issues on governance transfer as they re-centre their lives within and apart from their traditional family units with bioethical and legal considerations to navigate in their health and social care needs. It goes beyond this and includes areas that this growing population has never had to deal with, including the impact of climate change on care needs, which may impact on where they choose or need to live and other wider societal issues that can impact on their identity development for example sexual health issues, peer support, ongoing education and career progression.

The health of the next generation deserves better. This lack of engagement with realising the specific agency of the child and adolescent is in direct conflict with Article 12 of the UN Convention on the Rights of the Child (32) and Article 7 of the UN Convention of the Rights of Persons with Disabilities (33). *Article 12* states that children have the right to form and express views freely in all matters affecting them and that the views of the child must be given due weight in accordance with their age and maturity; and *Article 7* states that young people with disabilities have the right to express their views freely and should be provided with assistance where needed to realise that right. While the current EPW [pg 10] states that it “reflects WHO/Europe's determination to leave no one behind” and that it “puts strong emphasis on *leaving no one behind*”, it seems to have done just that, by not giving an explicit voice to the least heard groups in society.

To address current and emerging health problems in children and adolescents adequately and timely, this young population should have an explicit and prominent place in WHO's EPW. Not only does the WHO Regional Office for Europe have the responsibility to support all citizens in its Member States, WHO Europe also has the capacity and expertise to help improve child and adolescent public health. In addition, the WHO Regional Office for Europe, especially in lower income countries, has the influence necessary to drive significant improvements in child and adolescent health. WHO Europe can serve as a template for country action, stimulating national strategies and bringing people and actions together. We see and appreciate the strength and value of WHO Europe. It is a missed opportunity not to let children benefit from this, as children are our future.

## Data Availability

The original contributions presented in the study are included in the article, further inquiries can be directed to the corresponding author.

## References

[1] The European programme of work, 2020–2025: United action for better health. Copenhagen: WHO Regional Office for Europe (2021). Licence: CC BY-NC-SA 3.0 IGO.

[2] World Health Organization. Regional Office for Europe. Health 2020: a European policy framework supporting action across government and society for health and well-being (short version). World Health Organization. Regional Office for Europe (2013). Available at: https://apps.who.int/iris/handle/10665/131300

[3] World Health Organization. Investing in children: the European child and adolescent health strategy 2015–2020. Copenhagen: WHO Regional Office for Europe (2014). Available at: http://www.euro.who.int/__data/assets/pdf_file/0010/253729/64wd12e_InvestCAHstrategy_140440.pdf?ua=1

[4] World Health Organization. Children and adolescents in the WHO European Region (2019). Available at: https://www.who.int/europe/news-room/fact-sheets/item/children-and-adolescents-in-the-who-european-region (Accessed March 3, 2023).

[5] World Health Organization. The European health report 2021. Taking stock of the health-related sustainable development goals in the COVID-19 era with a focus on leaving no one behind. Copenhagen: WHO Regional Office for Europe (2022).

[6] UN Statistics Wiki. Indicator 3.2.1 (2018). Available at: https://unstats.un.org/wiki/display/SDGeHandbook/Indicator+3.2.1 (Accessed March 3, 2023).

[7] UNICEF Europe and Central Asia Regional Office. A review of the school-aged children & adolescent nutrition in Europe and Central Asia region. Geneva: United Nations Children’s Fund (UNICEF) (2020).

[8] Carrido-Miguel M Martínez-Vizcaíno V Oliveira A Martínez-Andrés M Sequí-Domínguez I Prevalence and trends of underweight in European children and adolescents: a systematic review and meta-analysis. Eur J Nutr. (2021) 60:3611–24. 10.1007/s00394-021-02540-0 33779808

[9] WHO European regional obesity report 2022. Copenhagen: WHO Regional Office for Europe (2022). Licence: CC BY-NC-SA 3.0 IGO.

[10] Tsur AM Twig G. The actual burden of obesity-accounting for multimorbidity. Lancet Diabetes Endocrinol. (2022) 10(4):233–4. 10.1016/S2213-8587(22)00073-0 35248170

[11] World Health Organization. Regional Office for Europe. Spotlight on adolescent health and well-being. Findings from the 2017/2018 Health Behaviour in School-aged Children (HBSC) survey in Europe and Canada. International report. Volume 2. Key data. World Health Organization. Regional Office for Europe (2020). Available at: https://apps.who.int/iris/handle/10665/332104. License: CC BY-NC-SA 3.0 IGO.

[12] Goto R Okubo Y Skokauskas N. Reasons and trends in youth’s suicide rates during the COVID-19 pandemic. Lancet Reg Health West Pac. (2022) 27:100567. 10.1016/j.lanwpc.2022.100567 35966624 PMC9366131

[13] UNICEF. The state of the world’s children 2021. On my mind. Promoting, protecting and caring for children’s mental health. United Nations Children’s Fund (UNICEF) (2021).

[14] Park M Budisavljević S Alemán-Díaz AY Carai S Schwarz K Kuttumutatova A Child and adolescent health in Europe: towards meeting the 2030 agenda. J Glob Health. (2023) 13:04011. 10.7189/jogh.13.04011 36655877 PMC9850873

[15] Rémy V Zöllner Y Heckmann U. Vaccination: the cornerstone of an efficient healthcare system. J Mark Access Health Policy. (2015) 3. 10.3402/jmahp.v3.27041 PMC4802703 27123189

[16] Ekezie W Awwad S Krauchenberg A Karara N Dembiński Ł Grossman Z Access to vaccination among disadvantaged, isolated and difficult-to-reach communities in the WHO European region: a systematic review. Vaccines. (2022) 10:1038. 10.3390/vaccines10071038 35891201 PMC9324407

[17] United Nations. Regional Information Center for Western Europe. Europe: one in three children overweight or obese (2022). Available at: https://unric.org/en/europe-one-in-three-children-overweight-or-obese/ (Accessed March 3, 2023).

[18] Baauw A Kist-van Holthe J Slattery B Heymans M Chinapaw M van Goudoever H (2019) Health needs of refugee children identified on arrival in reception countries: a systematic review and meta-analysis BMJ Paediatr Open 3:e000516. 10.1136/bmjpo-2019-000516 PMC6782036 31646192

[19] United Nations, Department of Economic and Social Affairs, Population Division. World population prospects 2019. Online edition, rev. 1 (2019). Available at: https://population.un.org/wpp/ (Accessed November 5, 2022).

[20] World Health Organization. Report of the commission on ending childhood obesity. Geneva: World Health Organization (2016).

[21] Ozawa S Clark S Portnoy A Grewal S Brenzel L Walker DG. Return on investment from childhood immunization in low- and middle-income countries, 2011–20. Health Aff. (2016) 35(2):199–207. 10.1377/hlthaff.2015.1086 26858370

[22] Sim SY Watts E Constenla D Brenzel L Patenaude BN. Return on investment from immunization against 10 pathogens in 94 low- and middle-income countries, 2011–30. Health Aff. (2020) 39(8):1343–53. 10.1377/hlthaff.2020.00103 32744930

[23] The decade of vaccine economics (DoVE). Available at: https://immunizationeconomics.org/dove-fs (Accessed November 14, 2022).

[24] Patton GC Sawyer SM Santelli JS Ross DA Afifi R Allen N Our future: a lancet commission on adolescent health and welfare. Lancet. (2016) 387:2423–78. 10.1016/S0140-6736(16)00579-1 27174304 PMC5832967

[25] Zhang Y Chai P Huang X Wan Q Tian X Guo F Financing adolescent health in China: how much, who pays, and where it goes. J Adolesc Health. (2020) 67:S38–47. 10.1016/j.jadohealth.2020.03.033 33246532

[26] Kessler RC Berglund P Demler O Jin R Merikangas KR Walters EE. Lifetime prevalence and age-of-onset distributions of DSM-IV disorders in the national comorbidity survey replication. Arch Gen Psychiatry. (2005) 62:593–602. 10.1001/archpsyc.62.6.593 15939837

[27] Stelmach R Kocher EL Kataria I Jackson-Morris AM Saxena S Nugent R. The global return on investment from preventing and treating adolescent mental disorders and suicide: a modelling study. BMJ Glob Health. (2022) 7(6):e007759. 10.1136/bmjgh-2021-007759 35705224 PMC9240828

[28] Michaud PA Visser A Vervoort JPM Kocken P Reijneveld SA Jansen DEMC. Availability and accessibility of primary mental health services for adolescents: an overview of national recommendations and services in EU. Eur J Public Health. (2020) 30(6):1127–33. 10.1093/eurpub/ckaa102 32820338

[29] Michaud PA Visser A Vervoort J Kocken P Reijneveld S Blair M Do European union countries adequately address the healthcare needs of adolescents in the area of sexual reproductive health and rights? Arch Dis Child. (2020) 105(1):40–6. 10.1136/archdischild-2019-317073 31270093 PMC6951236

[30] Michaud PA Jansen D Schrier L Vervoort J Visser A Dembiński Ł. An exploratory survey on the state of training in adolescent medicine and health in 36 European countries. Eur J Pediatr. (2019) 178(10):1559–65. 10.1007/s00431-019-03445-1 31463767 PMC6733827

[31] Brenner M Doyle A Begley T Doyle C Hill K Murphy M. Enhancing care of children with complex healthcare needs: an improvement project in a community health organisation in Ireland. BMJ Open Quality. (2021) 10:e001025. 10.1136/bmjoq-2020-001025 33619077 PMC7903071

[32] UNICEF. Convention on the rights of the child. Available at: https://www.unicef.org/child-rights-convention (Accessed March 3, 2023).

[33] United Nations. Department of Economic and Social Affairs. Convention on the rights of persons with disabilities (CRPD) (2022). Available at: https://www.un.org/development/desa/disabilities/convention-on-the-rights-of-persons-with-disabilities.html (Accessed March 3, 2023).

